# Familial (ATTR) amyloidosis misdiagnosed as the primary (AL) variant: a case report

**DOI:** 10.1186/1757-1626-2-9295

**Published:** 2009-12-09

**Authors:** Philip B Dattilo

**Affiliations:** 1 Division of Cardiology, University of Colorado, Denver, Denver, CO, USA

## Abstract

**Introduction:**

Primary (AL) Amyloidosis is arguably the most recognizable variant of the disease with many classic signs. However, it has been argued that the Familial variant (ATTR) is actually more prevalent. It is less recognizable, however, as its spectrum of organ involvement is frequently much more limited. The two variants carry significantly different prognoses, have divergent treatment strategies, and very different implications for the family members of patients. There is now a small amount of data that would suggest Familial Amyloidosis may be misdiagnosed as the AL form 2-4% of the time as a result of laboratory error.

**Case presentation:**

Herein a case of Familial Amyloidosis initially mistaken for the AL form based on a false positive laboratory result is presented. This case illustrates the high index of suspicion required for proper diagnosis of this rare disease.

**Conclusion:**

Clinician awareness of the various forms of Amyloidosis and the potential for lab error is key to ensuring an accurate diagnosis. The two most common forms carry significantly different implications for treatment and for potential impact on relatives. A high index of suspicion is required particularly for the Familial form of Amyloidosis.

## Introduction

Familial (ATTR) Amyloidosis is possibly the most common form of a rare disease, a result of mutant transthyretin deposition in the affected organs [1]. A specific variant of the transthyretin protein - Ile 122 - has been implicated as a cause of cardiac amyloidosis in those of African heritage [2]. It is believed to play a role in the increased rate of isolated cardiac amyloidosis seen in American Blacks after the age of 60. Isolated cardiac amyloidosis due to Primary (AL) Amyloidosis is relatively uncommon in general [1], and specifically in non-Caucasian populations [3-5]. Case series have described AL amyloidosis as being more prevalent in males and as occurring at a younger age than transthyretin-related amyloidosis [6,7]. AL amyloidosis commonly involves multiple organ systems, and not infrequently causes the nephrotic syndrome, the carpal tunnel syndrome, hepatomegaly, and macroglossia [6-8]. While ATTR amyloidosis may be more prevalent than AL, it is arguably less recognizable and therefore harder to diagnose, as it not infrequently presents simply as hypertrophic heart failure [1]. Given the difference in prognosis (ATTR carries a less rapidly lethal outcome), and the fact that ATTR is hereditary and potentially treatable if caught at an early stage by liver transplant [9] or possibly pharmacologically [10], it is important to differentiate these two types of amyloidosis.

The case below describes a case of ATTR Amyloidosis misdiagnosed as AL initially, based on a falsely positive stain for light chain deposition.

## Case presentation

A 75 year old Jamaican Female of African descent was admitted for shortness of breath. She had been complaining of fatigue and shortness of breath for six months since arriving in the U.S. from Jamaica. She reported mild symptoms in Jamaica, but felt they had worsened during her time in the States. She had been admitted multiple times to at least four different hospitals since her symptoms began. She had been treated for both congestive heart failure (CHF) and asthma exacerbations, despite carrying no significant history of either condition. At the time of the admission described herein, she had been discharged from another hospital two days prior on a tapered prednisone regimen prescribed for an "asthma exacerbation".

A review of systems at the time of admission revealed symptoms consistent with New York Heart Association Class IV CHF, including orthopnea, paroxysmal nocturnal dyspnea, lower extremity edema, and a virtually non-existent exercise tolerance.

On examination the patient was in acute distress, sitting bolt upright in bed and tripodding. She was obese. Jugular venous distension was difficult to discern due to her body habitus. She did not have a palpable point of maximal impulse. Her heart sounds were distant but regular with a II/VI systolic ejection murmur heard loudest over the right upper sternal border which did not radiate. Her breath sounds were absent at the bases and diminished 2/3 of the way up the posterior lung fields. She had 2+ pitting edema to the hips. There was no hepatomegaly or macroglossia.

**Figure 1:**
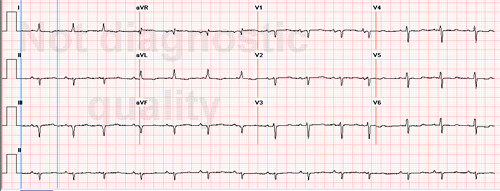
**The patient's ECG demonstrates a classic low voltage pattern**.

**Figure 2:**
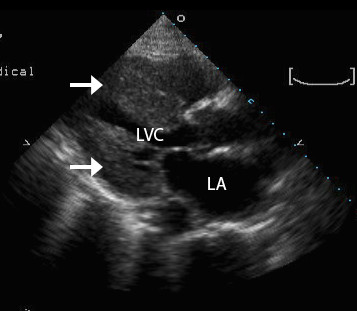
**An Echocardiogram shows dramatic left ventricular hypertrophy (arrows) and a small ventricular cavity**. LA - Left Atrium; LVC - Left Ventricular Cavity.

Her electrocardiogram (ECG) at the time of admission showed diffuse low voltage and Q waves in leads III and aVf (Figure 1). A trans-thoracic echocardiogram (TTE) was performed soon after admission (Figure 2). It showed moderately reduced left ventricular function with an ejection fraction (EF) of 33%, a small left ventricular cavity (20.4 cm^2 ^in diastole, 16.3 cm^2 ^in systole), markedly increased wall thickness (septal diameter in diastole = 1.86 cm), and severe concentric hypertrophy. Additionally, there was moderate mitral regurgitation. A speckled pattern in the myocardium was noted.

Both serum and urine protein electrophoresis demonstrated a normal protein distribution without a monoclonal spike. She had modestly impaired renal function (estimated GFR 51 mL/min by Cockocroft-Gault, 52 mL/min by Modification of Diet in Renal Disease Criteria) with no proteinuria.

She was diuresed using furosemide, and treated with monopril, metoprolol (later changed to carvedilol for better blood pressure control), and digoxin. There was little subjective or objective improvement in her condition with this treatment. The digoxin was stopped on the 5^th ^hospital day, as the suspicion for Amyloidosis at that point in time was high.

She underwent a fat pad aspiration biopsy, which was negative for amyloid by Congo Red Staining. At that time, it was felt that her refractory condition, in combination with the ECG and TTE data was sufficiently suggestive of amyloid disease that she should undergo endomyocardial biopsy as a means of definitive diagnosis. The biopsy revealed AL Amyloidosis with kappa and lambda light chain deposits. Stains for iron and amyloid AA were negative.

## Discussion

On further review, it was felt that the presentation was not particularly consistent with AL Amyloidosis given her demographics and the absence of other organ involvement or an elevated Ig fraction, and so the biopsy was sent for further analysis of transthyretin deposition. The results of that test were positive. Given the demographics and pertinent findings, it was concluded that the AL Amyloidosis finding was a false positive, and that the patient in reality had ATTR amyloidosis. Unfortunately, the patient had returned to Jamaica and was lost to follow-up before the correct diagnosis was made.

## Conclusion

Lab error with respect to differentiating Primary (AL) from Familial (ATTR) amyloidosis is uncommon, but not unheard of [2,11]. The largest series addressing Familial Amyloidosis noted a very low frequency of transthyretin amyloid samples cross reacting with light chain staining. The incidence was one in 50 samples - the one sample "presumably reflecting nonspecific binding due to variability in tissue processing" [2]. Lachmann et al [9] evaluated 350 cases of "AL" Amyloidosis in a British cohort, of which nearly 10% had been misdiagnosed. Thirteen of those cases (4%) were determined to be ATTR Amyloid using genetic analysis; 3 of those as a result of the Ile 122 variant.

This case was meaningful in that it highlights the importance of recognizing the disparate clinical characteristics of the various types of Amyloidosis. Further, it emphasizes the high level of suspicion needed to make a proper diagnosis. Given the rarity of the disease, many practitioners are not necessarily well aware of the differences. However, the distinction is critical. The prognosis varies significantly, along with the potential treatment strategy (chemotherapy for AL amyloid, liver transplant or possibly Non-Steroidal Anti-Inflammatory Drugs for ATTR). Further, the ability to anticipate disease in family members of ATTR Amyloid patients could help reduce the burden of this frequently terminal disease. Clinician awareness of the various forms of Amyloidosis and the potential for lab error is key to ensuring an accurate diagnosis and timely intervention.

## Consent

Multiple attempts were made to contact the patient regarding her condition, as well as to obtain consent for this case report. Unfortunately, she was only transiently in the U.S., and no forwarding information was left on her departure. Attempts to contact her family have been unsuccessful. After reading through the provisions in your instructions in the case where consent is unobtainable, I believe all three of the following conditions have been met:

• all reasonable attempts to gain consent have been made

• the patient is anonymous

• there is no reason to think that the patient or their family would object to publication

## Competing interests

The author declares that they have no competing interests.
